# A Frailty‐Based Plasma Proteomic Signature Capturing Overall Health and Well‐Being in Older Adults

**DOI:** 10.1111/acel.70144

**Published:** 2025-08-04

**Authors:** Sanish Sathyan, Fangyu Liu, Toshiko Tanaka, Luigi Ferrucci, Emmeline Ayers, B. Gwen Windham, Tina Gao, Erica F. Weiss, Julián Candia, Josef Coresh, Nir Barzilai, Sofiya Milman, Keenan A. Walker, Joe Verghese

**Affiliations:** ^1^ Department of Neurology Albert Einstein College of Medicine Bronx New York USA; ^2^ Department of Neurology Renaissance School of Medicine Stony Brook New York USA; ^3^ Laboratory of Behavioral Neuroscience National Institute on Aging Baltimore Maryland USA; ^4^ Longitudinal Studies Section Translational Gerontology Branch, National Institute on Aging Baltimore Maryland USA; ^5^ Department of Medicine MIND Center, University of Mississippi Medical Center Jackson Mississippi USA; ^6^ Institute for Aging Research, Department of Medicine Albert Einstein College of Medicine Bronx New York USA; ^7^ Institute for Optimal Aging New York University Grossman School of Medicine New York New York USA; ^8^ Department of Genetics Albert Einstein College of Medicine Bronx New York USA

**Keywords:** frailty, proteomic frailty index, proteomics, SomaScan assay

## Abstract

Frailty is an age‐related syndrome characterized by an increased vulnerability to adverse health outcomes in the face of stressors. By deriving a blood‐based proteomic signature for frailty, the current study aimed to enhance the understanding of frailty biology and created a person‐specific predictor for the risk of frailty and other adverse age‐related health outcomes. A 25‐protein signature (proteomic frailty index [pFI]) predictive of the cumulative frailty index (FI) in the LonGenity cohort was derived using a penalized regression method. The pFI was significantly correlated with the FI at baseline (Pearson *r* = 0.58) and showed significant associations with age‐related chronic conditions, incident mortality, and clinical measures. In an independent cohort of 5195 participants in the Atherosclerosis Risk in Communities study, pFI was successfully validated with measured FI (*r* = 0.61, *p* < 0.001) and was associated with physical frailty at baseline (*p* < 0.001). The pFI was significantly associated with physical, clinical, and cognitive measures, as well as incident mortality (HR [95% CI] = 1.13 [1.12–1.14]) and dementia (HR [95% CI] = 1.07 [1.05–1.09]) after accounting for demographic factors. The pFI was further validated against FI (*r* = 0.45, *p* < 0.001) in a second independent study in 654 participants from the Baltimore Longitudinal Study of Aging. In conclusion, we identified and validated a 25‐protein signature as an index of frailty that also captures overall well‐being, health, and risk for key age‐related diseases.

## Graphical abstract

We derived a 25‐protein blood‐based frailty signature (proteomic frailty index, pFI) predictive of the cumulative frailty index. The pFI was validated across independent cohorts and was significantly associated with physical, clinical, and cognitive function, as well as incident mortality and dementia, highlighting its potential as a biomarker of overall health and aging.

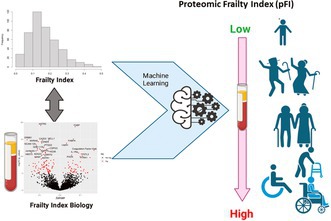

## 1 Introduction

Frailty is a complex late‐life phenotype characterized by cumulative declines across multiple physiological systems, and increasing vulnerability to adverse outcomes such as disability, hospitalization, and mortality (Fried et al. 2001; Rockwood et al. 2005). The complexity of frailty has made it challenging to elucidate its biology and to identify robust blood‐based biomarkers for frailty or frailty risk. With the advent of large‐scale proteomic platforms such as the SomaScan and Olink assays, rapid progress has been made in recent years to decipher proteomic changes associated with frailty (Landino et al. 2021; Liu et al. 2024; Sathyan, Ayers, Gao, Barzilai, et al. 2020). These studies identified various novel proteins and pathways associated with frailty, as assessed using multiple definitions, including the cumulative deficit model and physical frailty definitions (Landino et al. 2021; Liu et al. 2023; Liu et al. 2024; Sathyan, Ayers, Gao, Barzilai, et al. 2020). Our previous large scale proteomic analysis in LonGenity identified 143 proteins associated with the frailty index, highlighting pathways involved in lipid metabolism, musculoskeletal development and function, cell signaling, and cellular assembly and organization as being linked to frailty (Sathyan, Ayers, Gao, Barzilai, et al. 2020). The same study used an elastic net regression method to identify a 110‐protein frailty prediction model–which correlated strongly with measured frailty index (FI, *r* = 0.58).

Advancements in next‐generation sequencing and genotyping have accelerated frailty research, yet progress remains limited due to the complexity and low heritability of the phenotype (Atkins et al. 2021; Sathyan and Verghese 2020). Other methodologies, including transcriptomic and epigenetic analyses, have offered valuable insights; however, a complete understanding of the biology of frailty continues to remain elusive including deriving frailty signature (Li et al. 2022; Prince et al. 2019). For example, a study which derived a DNA methylation‐based frailty algorithm (the epigenetic frailty risk score [eFRS]) showed a 0.24 correlation with the FI (Li et al. 2022). This correlation was considerably lower than the 0.58 correlation we observed between the proteomic frailty and FI (Sathyan, Ayers, Gao, Barzilai, et al. 2020). This comparison highlights the superior predictive accuracy of proteomic predictor for frailty, likely due to its ability to capture a more dynamic and comprehensive profile of molecular alterations. Proteomic approaches for frailty prediction offer distinct advantages relative to other biomarker‐based approaches. For clinical utility as well as to understand key drivers of frailty, the derivation of a parsimonious index with fewer proteins is essential. It also enhances the feasibility and cost‐effectiveness of translating the biomarker into clinical or population‐level settings, while improving the robustness and interpretability of the index. To this end, we aimed to create a Proteomic Frailty Index (pFI) that can serve as a biological proxy for clinical frailty measured by FI, and that can capture the overall health of an individual. It may also be developed as a robust and versatile tool for comprehensive health evaluation, risk stratification for medical interventions, and for monitoring and prognosis, thereby improving patient care and outcomes in aging populations. Compared to traditional frailty indices that require assessing multiple clinical and functional components, the pFI would be a more practical option, especially in clinical settings. It overcomes key limitations of traditional physical frailty assessments, allowing application even in non‐ambulatory or cognitively impaired individuals. The pFI can be integrated into routine laboratory workflows and holds promises for early detection and longitudinal monitoring of health decline. Its scalability and potential for automation further enhance its suitability for widespread clinical implementation.

In this study, we employed a comprehensive approach to construct a proteomic signature of frailty (pFI) from frailty‐associated proteins in the LonGenity cohort. To establish the robustness of this newly derived construct, we conducted cross‐validation in two large, independent aging cohorts: the Atherosclerosis Risk in Communities (ARIC) study and Baltimore Longitudinal Study of Aging (BLSA). We carried out association analyses of the pFI with prevalent frailty to evaluate its concurrent validity. Frailty reflects dysfunction happening across multiple organ systems (Chen et al. 2014), and the pFI is derived from the cumulative frailty index, which is a multidimensional construct (Rockwood and Mitnitski 2007). Therefore, it is essential to demonstrate the predictive validity of the pFI across a wide range of health outcomes, each capturing health status in specific organ systems. To this end, we assessed the association of the pFI with a spectrum of health outcomes, including all‐cause mortality, chronic conditions, and various clinical, physical, and cognitive phenotypes, including incident dementia. This framework highlights the potential utility of the pFI as a biomarker for frailty, which can also provide insights into health trajectories in aging populations.

## 2 Results

### 2.1 Development of pFI in LonGenity Cohort

#### 2.1.1 LonGenity Cohort

Demographic and clinical characteristics are summarized in Table 1. Of the 880 eligible individuals with phenotype and proteomic data in the LonGenity cohort, the mean age of participants was 75.3 ± 6.6 years, 54.8% were women, and the average years of education was 17.52 ± 2.88 years. The mean FI score for the eligible study sample was 0.163 (SD = 0.086).

**TABLE 1:** Demographic and clinical characteristics of LonGenity cohort.

Variables	LonGenity
Participants, *n* (%)	880
Age, mean ± SD, years	75.3 ± 6.6
Women, *n* (%)	482 (54.8)
OPEL, *n* (%)	432 (49.1)
Education, mean, years	17.5 ± 2.9
Rockwood Frailty Index (mean ± SD)	0.16 ± 0.09
Medical illnesses	
Stroke, %	3.3
Diabetes, %	8.5
Myocardial infraction, %	5.8
Arthritis, %	41.6
Hypertension, %	43.5

#### 2.1.2 Construction of pFI and Association With FI

In the first step, association analysis adjusted for age, sex, and parental longevity had identified 143 proteins associated with prevalent frailty in the LonGenity cohort. A total of 440 participants were selected for the training dataset using random stratified sampling based on frailty distribution, while the remaining 440 participants were designated as the validation set.

**FIGURE 1:**
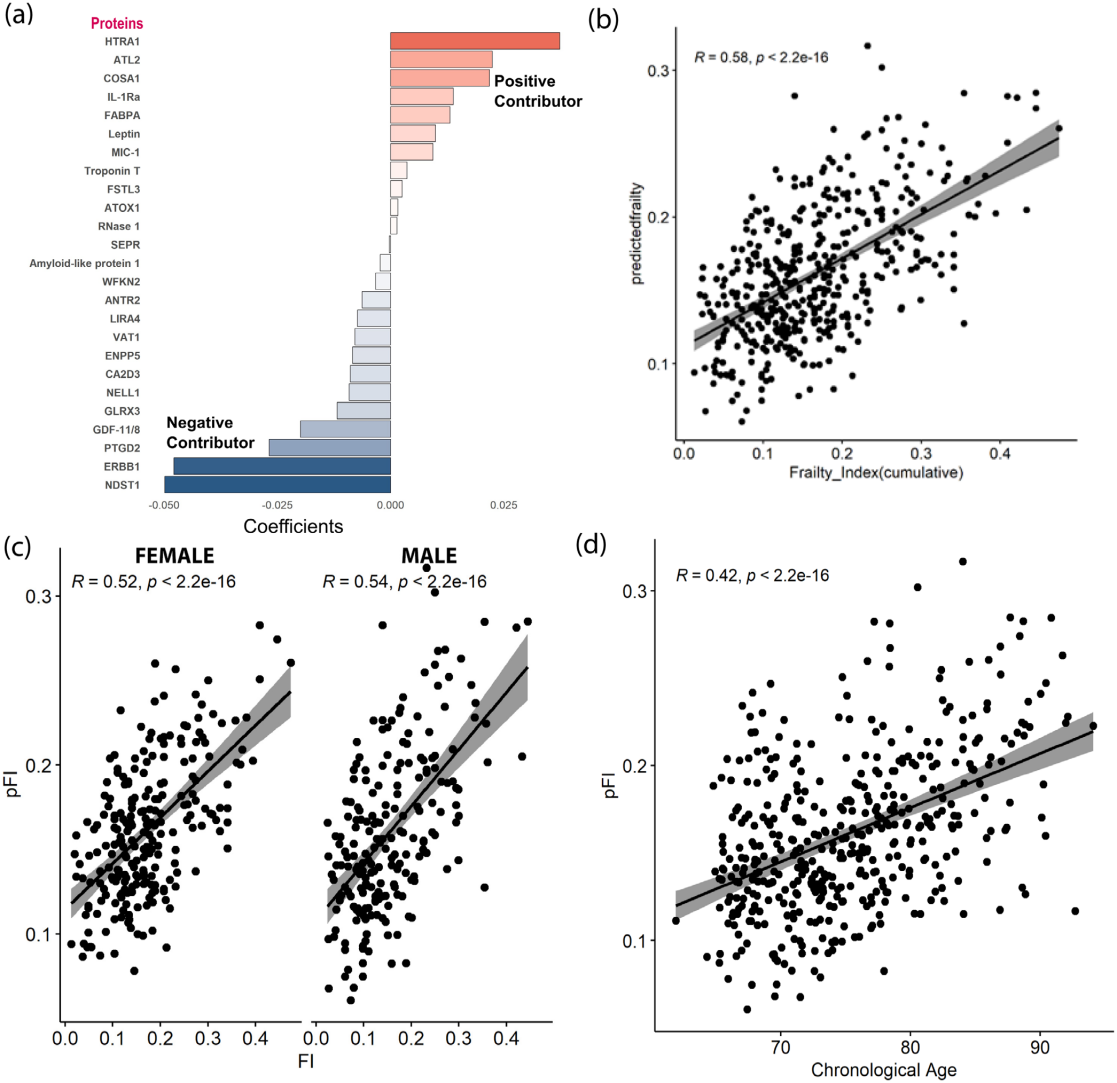
Proteomic Frailty Index. (a) Key proteins graphically depicted based on the contribution towards pFI. (b) Correlation of observed cumulative frailty index and predicted frailty index using proteomic data. Correlation of predicted frailty using proteomic markers and cumulative frailty index was 0.58. (c) Sex specific correlation showing similar level of Pearson correlation. (d) Correlation of pFI with chronological age.

To create a proteomic predictor of frailty (pFI), we fitted an elastic net regression model to select a parsimonious subset of the 143 proteins that best predicted the cumulative frailty index. The coefficients of the 25 proteins selected using elastic net regression are depicted in Figure 1a and detailed in Table 2. A detailed overview of the possible functionality of proteins with respect to aging and age‐associated phenotypes is provided in Table S1. The mean pFI score for the validation study sample was 0.161 (SD = 0.045). The Pearson correlation between pFI and the baseline FI was 0.58 in the validation set (Figure 1b). A similar correlation was observed in male and female (Figure 1c) and showed a positive correlation with chronological age (*r* = 0.42) (Figure 1d). The pFI showed a correlation of 0.52 with FI at Visit 2 and 0.51 with FI at Visit 3 among participants in the LonGenity cohort.

**TABLE 2:** Modeling frailty using the plasma proteome. 25 proteins used for the construction of frailty and its coefficient are provided.

SeqID	Target	Target full name	Coefficient
		(Intercept)	0.7113
15594‐47	HTRA1	Serine protease HTRA1	0.0373
6379‐62	ATL2	ADAMTS‐like protein 2	0.0224
10702‐1	COSA1	Collagen alpha‐1 (XXVIII) chain	0.0218
5353‐89	IL‐1Ra	Interleukin‐1 receptor antagonist protein	0.0138
15386‐7	FABPA	Fatty acid‐binding protein, adipocyte	0.0131
8484‐24	Leptin	Leptin	0.0099
4374‐45	MIC‐1	Growth/Differentiation factor 15	0.0093
5315‐22	Troponin T	Troponin T, cardiac muscle	0.0036
3438‐10	FSTL3	Follistatin‐related protein 3	0.0025
19233‐75	ATOX1	Copper transport protein ATOX1	0.0016
7211‐2	RNase 1	Ribonuclease pancreatic	0.0014
5029‐3	SEPR	Prolyl endopeptidase FAP	−0.0003
721025	Amyloid‐like protein 1	Amyloid‐like protein 1	−0.0023
3235‐50	WFKN2	WAP, Kazal, immunoglobulin, Kunitz and NTR domain‐containing protein 2	−0.0033
15559‐5	ANTR2	Anthrax toxin receptor 2	−0.0063
8299‐66	LIRA4	Leukocyte immunoglobulin‐like receptor subfamily A member 4	−0.0073
18175‐65	VAT1	Synaptic vesicle membrane protein VAT‐1 homolog	−0.0079
6556‐5	ENPP5	Ectonucleotide pyrophosphatase/phosphodiesterase family member 5	−0.0084
8885‐6	CA2D3	Voltage‐dependent calcium channel subunit alpha‐2/delta‐3	−0.0089
6544‐33	NELL1	Protein kinase C‐binding protein NELL1	−0.0092
16596‐25	GLRX3	Glutaredoxin‐3	−0.0118
2765‐4	GDF‐11/8	Growth/Differentiation factor 11/8	−0.0199
12549‐33	PTGD2	Hematopoietic prostaglandin D synthase	−0.0268
2677‐1	ERBB1	Epidermal growth factor receptor	−0.0478
6927‐7	NDST1	Bifunctional heparan sulfate N‐deacetylase/N‐sulfotransferase 1	−0.0498

#### 2.1.3 pFI and Mortality

**FIGURE 2:**
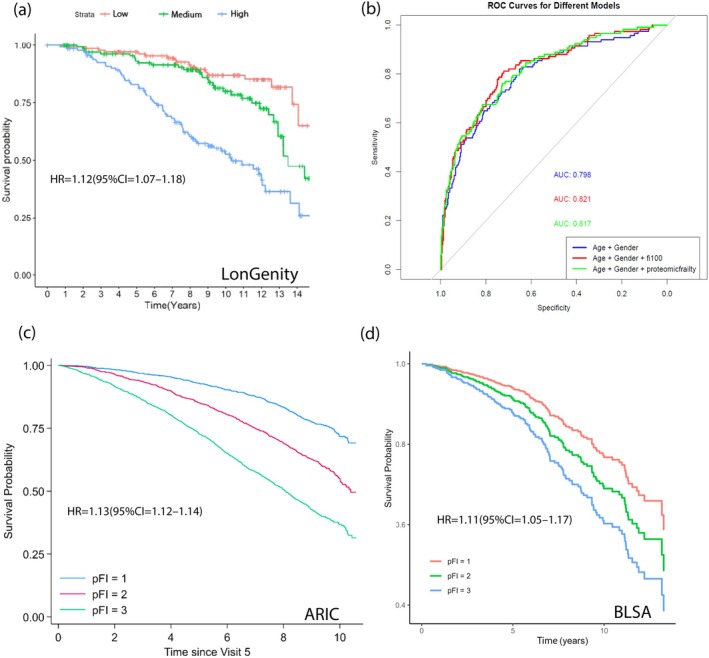
Proteomic Frailty Index and mortality. (a) Kaplan–Meier survival curves for time to death for LonGenity participants based on proteomic frailty index tertile (b) ROC curves for model containing pFI and FI with mortality. AUC of the pFI and FI model was 0.82 accounting for age and sex. (c, d) Kaplan–Meier survival curves for time to death for ARIC and BLSA participants respectively based on proteomic frailty index tertile. Model adjusted for age, sex, race‐center composite variable (ARIC) or race (BLSA).

In the validation arm of 440 participants in LonGenity cohort, 117 deaths were reported, with a mean follow‐up time of 8.1 ± 4.0 years. The pFI was associated with mortality with adjustment for chronological age and sex (HR = 1.12, 95% CI = 1.07–1.18, *p* = 2.25E−06). This association remained statistically significant after further adjustment for baseline frailty (HR = 1.09, 95% CI = 1.04–1.15, *p* = 7.34E−04). Participants in the uppermost pFI tertile (pFI > 0.174) were at over four times greater risk of incident death compared to those in the lowest pFI tertile (pFI < 0.137) (Figure 2a). Specifically, 68 deaths occurred among the 146 participants in the uppermost tertile (46.5%), whereas only 15 deaths were reported among the 143 participants in the lowest pFI tertile (10.4%). The pFI, derived from the proteome, demonstrated good and similar discriminatory ability for mortality (Age + Sex + pFI: AUC = 0.82), comparable to the FI (Age + Sex + FI: AUC = 0.82) (Figure 2b). When considered independently, without accounting for Age and Sex, the AUC for the FI was 0.72, and for the pFI, it was 0.73, indicating that both indices demonstrated similar stand‐alone discriminatory performance in relation to mortality (Figure S1).

#### 2.1.4 Association of pFI With Chronic Conditions and Clinical Measures

In the validation arm, the pFI was significantly associated with multiple age‐associated phenotypes at baseline, including diabetes (OR = 1.24, 95% CI = 1.15–1.35, *p* = 2.15E−07), hypertension (OR = 1.17, 95% CI = 1.11–1.24, *p* = 8.81E−09) and obesity (OR = 1.23, 95% CI = 1.16–1.32, *p* = 3.15E−11) (Table 3). Higher pFI was significantly associated with lower gait speed (*β* [SE] = −0.014 [−0.019, −0.009]; *p* = 6.81E‐07) and global cognition composite (*β* [SE] = −0.016–0.029, [−0.003]; *p* = 0.013), as well as higher levels of triglyceride(*β* [SE] =3.676 [2.476, 4.876]; *p* = 4.31E‐09) and insulin (*β* [SE] = 0.553 [0.355, 0.752]; *p* = 8.06E‐08). In contrast, higher pFI was associated with lower HDL (*β* [SE] = −1.182 [−1.535, −0.829]; *p* = 1.74E‐10) (Table 3).

**TABLE 3:** Association analysis of pFI with chronic condition, clinical, physical, and cognitive measure in LonGenity Cohort. All models were adjusted for age and sex.

Chronic condition	OR (95% CI)	*p*
Hypertension	1.17 (1.11–1.24)	8.81E‐09
Diabetes	1.24 (1.15–1.35)	2.15E‐07
MI	1.04 (0.95–1.14)	0.391
Cardiac irregularities	1.09 (0.99–1.22)	0.100
Stroke	1.06 (0.94–1.20)	0.342
Obesity	1.23 (1.16–1.32)	3.15E‐11
Depression	1.12 (1.05–1.19)	2.17E‐04
Physical inactivity	1.10 (1.05–1.17)	3.87E‐04
Osteoarthritis	1.07 (1.01–1.12)	0.011
Cancer	1.02 (0.97–1.07)	0.420

	*β* (95% CI)	*p*
Biological measures		
Cholesterol	−1.089 (−2.188, 0.011)	0.052
HDL	−1.182 (−1.535, −0.829)	1.74E‐10
Triglycerides	3.676 (2.476, 4.876)	4.31E‐09
Glucose	0.937 (0.461–1.412)	1.28E‐04
Insulin	0.553 (0.355, 0.752)	8.06E‐08
eGFR	−1.710 (−2.120, −1.300)	2.81E‐15
Clinical measures		
Systolic	0.556 (0.130, 0.982)	0.011
Diastolic	0.097 (−0.098, 0.291)	0.329
Grip strength	−0.266 (−0.521, −0.012)	0.040
Gait speed	−0.014 (−0.019, −0.009)	6.81E‐07
Peakflow	−4.332 (−7.357, −1.307)	0.005
Cognitive measures		
Language	−0.013 (−0.032, 0.006)	0.165
Executive	−0.023 (−0.045, −0.001)	0.045
Memory	−0.004 (−0.017, 0.009)	0.532
Attention	−0.035 (−0.053, −0.017)	1.92E‐04
VisuoMotor	−0.038 (−0.059, −0.017)	3.59E‐04
Global cognition composite	−0.016 (−0.029, −0.003)	0.013

### 2.2 Validation of pFI: ARIC and BLSA Study

Demographic and clinical characteristics of the ARIC participants are summarized in Table S2. The mean age of the participants was 75.7 ± 5.2 years, and 57% were female. The distribution of pFI is shown in Figure S2. The pFI was constructed using proteomic data from 5195 participants in the ARIC study. The pFI was strongly correlated with the FI (*n* = 5195, *r* = 0.61, *p* ≤ 0.05) (Figure S2).

Demographic and clinical characteristics of the BLSA participants are summarized in Table S3. The pFI was constructed using proteomic data from 654 participants in the BLSA study. The distribution of pFI is shown in Figure S3. The mean age of the participants was 76.9 ± 7.2 years, and 52.1% were female. The pFI correlated strongly with measured FI (*n* = 654, *r* = 0.45, *p* ≤ 0.05) (Figure S3).

#### 2.2.1 Association of pFI and Mortality in Validation Cohorts

In total 5164 older adults from the ARIC Study (Visit 5, 2011‐13; 99%) had proteomic measurement, complete covariates and follow‐up data for mortality. By December 31, 2021 (mean follow‐up time: 8.05 ± 2.47), 1757 deaths had occurred. Baseline (Visit 5) pFI was associated with mortality after adjustment for chronological age, sex, and race‐center composite variable (HR = 1.13, 95% CI = 1.12–1.14, *p* = 5.7E‐114). There were 885 deaths in the uppermost tertile group (51%), whereas only 319 deaths were reported in the lowest pFI tertile group (19%) (Figure 2c).

Of the 654 BLSA participants with proteomic measurements, complete covariates and follow‐up data for mortality, 129 deaths (22%) were documented between the visit of proteomic measurement and 2021 (mean follow‐up time: 6.285 ± 3.86 years). pFI was associated with mortality after adjustment for chronological age, sex, and race (HR = 1.11, 95% CI = 1.05–1.17, *p* = 2.2E‐04) (Figure 2d).

#### 2.2.2 Association of pFI With Physical Frailty Phenotype

**FIGURE 3:**
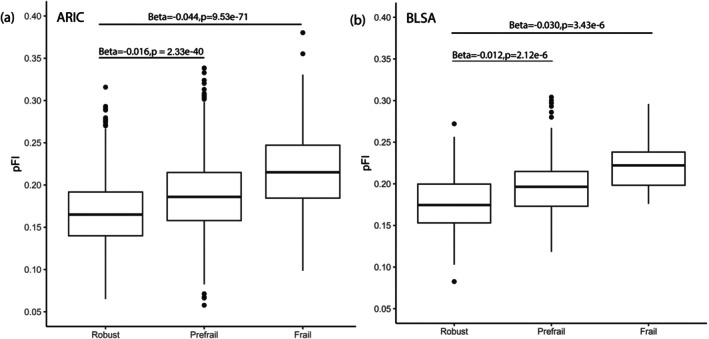
Association of pFI with prevalent physical frailty in ARIC (a) and BLSA (b) at baseline for the analysis.

Of the 5195 participants with a pFI score at Visit 5 in ARIC, 4722 had concurrent physical frailty measurements. There were significant cross‐sectional associations between pFI and frailty phenotype, where average pFI scores were 0.17 (SD = 0.04) for the robust category, 0.19 (SD = 0.04) for prefrail, and 0.22 (SD = 0.05) for frail. There was a significantly lower pFI in robust participants compared to prefrail (*β* = −0.016, SE = 0.001, *p* = 2.33E‐40) and frail (*β* = −0.044, SE = 0.002, *p* = 9.53E‐71) after adjusting for chronological age, sex, and race‐center composite variable (*N* = 4696) (Figure 3a). Similarly, in 647 BLSA participants, there was significant cross‐sectional association between pFI and frailty phenotype where the average pFI for robust, prefrail and frail categories were 0.18 (SD = 0.03), 0.20 (0.34), and 0.22 (0.03), respectively. There was significantly lower pFI in robust participants compared to prefrail (*β* = −0.012, SE = 0.003, *p* = 2.12E‐6) and frail (*β* = −0.030, SE = 0.006, *p* = 3.43E‐6) (Figure 3b).

Further, in both the ARIC and BLSA, pFI was negatively associated with grip strength (*β*_ARIC_ = −0.242, *p* = 1.60E‐22; *β*_BLSA_ = −0.205, *p* = 0.015) and gait speed (*β*_ARIC_ = −0.018, *p* = 1.83E‐162; *β*_BLSA_ = −0.017, *p* = 1.75E‐10), two measures of physical function included in the physical frailty phenotype (Table 4).

**TABLE 4:** Association analysis of pFI with chronic condition, clinical, physical and cognitive measure in the ARIC study (visit 5) and BLSA. All models were adjusted for age, sex and race‐center composite variable.

Chronic conditions	ARIC			BLSA		
Phenotype	*N*/Cases	OR (95% CI)	*p*	*N*/Cases	OR (95% CI)	*p*
Hypertension	5099/3749	1.14 (1.12, 1.16)	1.27E‐50	400/654	1.18 (1.11–1.25)	1.74E‐08
Diabetes	5021/1643	1.18 (1.16, 1.20)	1.32E‐90	91/654	1.20 (1.12–1.29)	3.25E‐07
CHD	5077/787	1.11 (1.09, 1.13)	1.05E‐29	73/654	1.21 (1.12–1.31)	1.21E‐06
Heart failure	5166/678	1.19 (1.16, 1.21)	1.88E‐63	46/654	1.25 (1.14–1.38)	6.45E‐06
Stroke	5159/190	1.10 (1.06, 1.13)	7.11E‐09	85/654	1.03 (0.96–1.11)	0.407

	ARIC			BLSA		
	*N*	*β* (95% CI)	*p*	*N*	*β* (95% CI)	*p*
Biological measures						
Cholesterol (SI unit)	5166	−0.058 (−0.065, −0.052)	1.03E‐65	651	−0.040 (−0.061, −0.019)	2.08E‐04
HDL (SI unit)	5166	−0.030 (−0.031, −0.028)	4.92E‐182	651	−0.046 (−0.055, −0.037)	2.63E‐21
Triglycerides (SI unit)	5166	0.035 (0.031, 0.039)	4.75E‐66	651	0.044 (0.032, 0.056)	1.23E‐12
Glucose (SI unit)	4951	0.042 (0.035, 0.049)	3.02E‐32	654	0.049 (0.036, 0.063)	1.69E‐12
Clinical measures						
Systolic BP	5139	−0.080 (−0.203, 0.044)	0.205	647	0.277 (−0.081, 0.635)	0.129
Diastolic BP	5139	−0.115 (−0.186, −0.044)	0.002	647	0.062 (−0.150, 0.273)	0.568
Grip strength	4757	−0.242 (−0.290, −0.194)	1.60E‐22	646	−0.205 (−0.370, −0.040)	0.015
Gait speed (m/s)	4737	−0.018 (−0.020, −0.017)	1.83E‐162	644	−0.017 (−0.022, −0.012)	1.75E‐10
Cognitive measures						
Language	5107	−0.030 (−0.035, −0.025)	1.29E‐31			
Executive function	5048	−0.040 (−0.044, −0.035)	3.18E‐62			
Memory	5109	−0.014 (−0.019, −0.009)	5.46E‐08			
Global	5119	−0.032 (−0.037, −0.027)	4.23E‐37			

#### 2.2.3 pFI Association With Chronic Medical Conditions and Clinical Measures

In ARIC, pFI was strongly associated with prevalent chronic medical conditions, including hypertension, diabetes, CHD, heart failure, and stroke (Table 4). Similarly, in the BLSA, pFI was strongly associated with prevalent chronic medical conditions including hypertension, diabetes, heart disease, heart failure (Table 4). However, there was no significant association with stroke (OR = 1.03, 95% CI = 0.96–1.11, *p* = 0.407) (Table 4).

Cross‐sectional associations between pFI and key physiological variables were observed as well. In both the ARIC and BLSA studies, pFI was positively associated with levels of glucose (*β*_ARIC_ = 0.042, *p* = 3.02E‐32; *β*_BLSA_ = 0.049, *p* = 1.69E‐12; Table 4) and triglycerides (*β*_ARIC_ = 0.035, *p* = 4.75E‐66; *β*_BLSA_ = 0.044, *p* = 1.23E‐12) while negatively associated with HDL (*β*_ARIC_ = −0.030, *p* = 4.92E‐182; *β*_BLSA_ = −0.046, *p* = 2.63E‐21) and total cholesterol (*β*_ARIC_ = −0.058, *p* = 1.03E‐65; *β*_BLSA_ = −0.040, *p* = 2.08E‐4).

#### 2.2.4 pFI Association With Cognitive Scores and Incident Dementia

**FIGURE 4:**
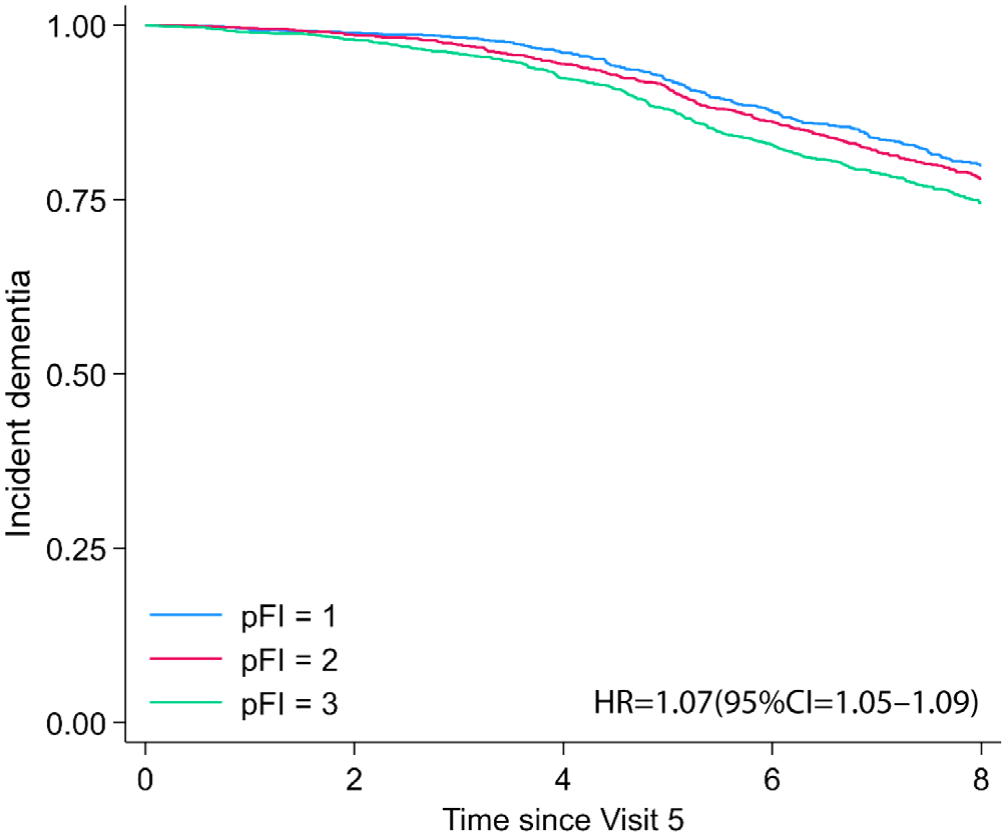
Proteomic Frailty Index and Dementia Kaplan–Meier survival curves for time to incident dementia for ARIC visit 5 participants based on proteomic frailty index tertile.

In ARIC, a higher pFI score was associated with lower global cognition (*β* = −0.032, *p* = 4.23E‐37) and reductions in performance on measures of language, executive, and memory functions in cross‐sectional analyses (Table 4). Additionally, higher pFI at baseline (Visit 5) was significantly associated with incident dementia after adjusting for age, sex and race‐center composite variable (HR = 1.07, 95% CI = 1.05–1.09, *p* = 9.34E‐18; 942 incident cases) over a mean follow‐up period of 7.22 years (SD = 1.82) (Figure 4).

## 3 Discussion

The present study characterizes pFI, a 25‐protein blood‐based biomarker of frailty. We demonstrated that pFI was (i) capable of estimating FI, and (ii) strongly associated with overall health outcomes including diverse age‐related disease phenotypes, physical and cognitive function, physiological and biological measures, and mortality. Our results suggest that the pFI has the potential to serve as a clinically translatable and biologically informative measure of frailty, as well as an indicator of overall well‐being in older adults. These findings were validated and extended to new dimensions in two independent cohorts, further strengthening the validity of this new construct.

This is the first comprehensive attempt to develop and validate a parsimonious proteomic signature of frailty. This study also sheds light on proteins that may play pivotal roles in frailty pathogenesis. The strongest positive contributor to pFI was Serine protease HTRA1, a secreted enzyme proposed to regulate the availability of insulin‐like growth factors (IGFs) by cleaving IGF‐binding proteins. While HTRA1 has been identified as a regulator of cell growth, the IGF pathways have been associated with longevity and frailty (Clegg and Hassan‐Smith 2018; Milman et al. 2014). HtrA1 also inhibits the signaling of TGF‐β proteins, which have anti‐inflammatory properties, and have been strongly associated with frailty in plasma using the ELISA method, further strengthening the evidence for its role in frailty (Lorenzi et al. 2016). N‐Deacetylase/N‐Sulfotransferase isoform 1 (NDST‐1) had the strongest negative loading on the pFI construct. NDST‐1 is a critical enzyme in the biosynthesis of heparan sulfate, a highly sulfated polysaccharide that plays important physiological roles. Loss of NDST1 N‐sulfation activity is associated with impaired cognitive functions, defective craniofacial development, delayed ossification, and impaired mandibular and temporomandibular joint development (Khosrowabadi et al. 2024; Walker et al. 2023; Whitelock and Melrose 2011). Other important proteins in the model include leptin and FABP‐A, which are involved in lipid metabolism; Troponin T, an important biomarker for heart muscle damage; GDF‐11, which acts as a rejuvenation factor (Ma et al. 2021); and IL1RA, which plays a crucial role in immune response and regulation of lipid and lipoprotein metabolism through direct actions on adipose tissues (Matsuki et al. 2003). The diverse biology of the proteins included in the model likely accounts for pFI's ability to capture the overall health of the participants while also pointing towards key drivers of frailty. Pathway analysis of all associated proteins previously identified pointed towards lipid metabolism, musculoskeletal development and function, cell signaling, and cellular assembly and organization as key pathways linked to frailty (Sathyan, Ayers, Gao, Milman, et al. 2020). Considering the diverse functionality of the smaller subset of selected proteins, which map to a wide range of biological pathways, we did not focus on drawing pathway‐based conclusions that may not be appropriate for a complex phenotype like frailty.

In this study, we demonstrate that the proteomic frailty index (pFI) is significantly associated with mortality, multiple chronic conditions, and various physical and cognitive measures in older adults, highlighting its potential as a robust marker of frailty and overall health status. Similar to the frailty phenotype, which has been shown to predict hospitalization, disability, and mortality (Fried et al. 2001), the pFI is also associated with major clinical outcomes including diabetes, hypertension, and cardiovascular disease. Moreover, the pFI correlates strongly with objective physical measures such as gait speed and grip strength—core components of the frailty phenotype—further supporting its utility in capturing physical frailty and functional decline. These findings position the pFI as a promising tool for identifying individuals at risk of adverse health outcomes in aging populations (Fried et al. 2001). This comprehensive analysis also demonstrated that the pFI was associated with a broad set of risk and protective factors linked to health and aging. For example, higher pFI was strongly associated with higher triglyceride levels in all the analyses, whereas HDL showed an inverse association with pFI, consistent with HDL's protective effect. This, along with the contribution of markers like leptin, FABP‐A, and IL1RA in the prediction model, underscores the potentially important role of lipid metabolism pathways in frailty pathogenesis and overall health. The association of pFI with a wide range of disease risk factors supports the use of this index as a biomarker to track numerous domains of aging. Consistent with the concept of FI as a marker of accumulation of health deficits (Rockwood and Mitnitski 2007), the proteomic index could capture health status across different domains from a single sample of blood. Future studies should explore whether this proteomic index can capture changes in disease risk factor profiles in response to an intervention.

The pFI was also associated directly with the physical frailty phenotype in the study. Further, pFI was predictive of all‐cause mortality in all three cohorts, underscoring the utility of this molecular construct as a predictor of health span. Individuals with pre‐frailty and frailty were at higher risk of dementia incidence (Petermann‐Rocha et al. 2020; Ward et al. 2025), and frailty has been identified as a moderator of the relationship between neuropathology and dementia (Wallace et al. 2019). Here, we show that, like frailty, pFI is also strongly associated with incident dementia and cognitive function, suggesting that, at the proteomic level, frailty and dementia may have overlapping biology. Our earlier study has also shown frailty to be associated with predementia syndrome‐motoric cognitive risk syndrome‐ in the same cohort (Sathyan et al. 2019). These findings also reinforce the concept of cognitive frailty, characterized by the simultaneous presence of physical frailty and cognitive impairment in the absence of dementia (Kelaiditi et al. 2013), and highlight the potential of pFI as a molecular signature of this high‐risk state. Further studies are warranted to validate and expand upon these observations.

This study has several strengths, including the use of a large‐scale proteomic panel and successful validation and further analysis in an independent cohort. The strong association with both cumulative and physical frailty definitions makes it a unique marker for determining frailty risk. The ability to capture frailty was further validated by the strong association of this construct with grip strength and gait speed—two key domains of physical frailty measurement. Using a two‐stage approach, we developed a concise 25‐protein signature that will be easy to establish and validate in future studies. Although derived in a homogeneous cohort of Ashkenazi Jewish participants, the success of pFI validation in a different cohort of Black and White adults suggests robustness and potential applicability across diverse populations, indicating that it may serve as a reliable tool for assessing frailty and related health outcomes in varied demographic groups. A major strength of this study is the use of a uniform proteomic platform—SomaScan assay—across all three cohorts, which enabled consistent measurement of protein levels and reduced technical variability. This methodological consistency enhances the comparability of results across cohorts and strengthens the validity of the proteomic frailty index (pFI) as a robust and generalizable measure. However, limitations include unexplained biology and the possibility of more accurate biomarkers among proteins not covered in the proteomic panels and associated with frailty. Although all‐cause mortality was consistently defined across cohorts, variations in follow‐up intensity, data sources, and the end dates of mortality tracking may introduce heterogeneity in the completeness and timing of mortality ascertainment. It is important to note that most large‐scale proteomic studies investigating the biology of frailty using the SomaScan assay have been conducted within these three cohorts (Landino et al. 2021; Liu et al. 2023; Liu et al. 2024; Sathyan, Ayers, Gao, Milman, et al. 2020). This underscores the need for replication and validation of the proteomic frailty signature in independent cohorts across diverse geographical and demographic populations. Future studies should aim to replicate and independently derive the pFI using other proteomic technologies, such as Olink or mass spectrometry, to ensure broader applicability and robustness. We also acknowledge the limitations of our study related to variability in parameters across cohorts and heterogeneity in data collection methods. These differences stem from the unique design and focus of each cohort, which, while offering valuable strengths, contribute to differences in the phenotypic data captured. While this study provides a preliminary framework for deriving a proteomic signature that captures aspects of overall health, further research is needed to validate these findings and to develop more robust and novel predictors using advanced methodologies and evolving proteomic platforms. Additionally, longitudinal analyses are needed to better understand the association between pFI and incident health outcomes.

In conclusion, this study derived and validated a proteomic frailty index (pFI), a novel construct, and demonstrated its potential as an indicator of frailty, overall health, and aging, including mortality, chronic conditions, physical frailty, and cognitive decline. Future studies should continue exploring the utility of pFI in diverse populations and investigate its potential to inform targeted interventions for aging‐related health risks. Looking ahead, we envision the pFI as a potential screening tool for the early identification of individuals at increased risk of frailty‐related adverse outcomes, particularly among older adults and those with multimorbidity. In clinical settings, the pFI may support risk stratification and guide personalized preventive interventions, ultimately contributing to enhanced healthspan and more efficient healthcare delivery.

## 4 Methods

### 4.1 Study Population

In this study, we utilized proteomics data from three cohorts analyzed using the SomaScan assay. In the training step, the proteomic predictor was derived using participants from the LonGenity cohort (*n* = 440), based on a subset of 143 proteins that we reported was associated with the FI (Sathyan, Ayers, Gao, Barzilai, et al. 2020). The pFI was computed for 440 participants in the validation arm of the LonGenity cohort, as well as for approximately 5195 older adults in the ARIC study and 654 older adults in the BLSA.

#### 4.1.1 LonGenity Cohort

The LonGenity study, established in 2007, recruited a cohort of Ashkenazi Jewish (AJ) adults aged 65 and older. Participants were categorized into two groups: offspring of parents with exceptional longevity (OPEL), defined as having at least one parent who lived to age 95 or older, and offspring of parents with usual survival (OPUS), defined as having neither parent survive to age 95. The study's goal is to identify genotypes and phenotypes associated with longevity and successful aging. Participants were systematically recruited using public records, as well as through contacts at synagogues, community organizations, and advertisements in Jewish newspapers in the New York City area. Potential participants were contacted by telephone to assess their interest and eligibility. Exclusion criteria include the following: a score > 8 on the Blessed Memory‐Information‐Concentration test and > 2 on the ad8 (Galvin et al. 2006) at the initial screening interview, severe visual impairment, and having a sibling in the study. At baseline and during annual follow‐up visits, participants underwent a detailed medical history evaluation and cognitive testing. All participants provided written informed consent for study assessments and genetic testing prior to enrollment. The study protocol was approved by the Albert Einstein College of Medicine Institutional Review Board. During annual visits, participants completed neuropsychological tests evaluating memory, language, visuospatial functioning, attention, and executive function under the supervision of the study neuropsychologist. Global cognition was assessed with a standardized composite score generated from relative performance of the subject in the Free and Cued Selective Reminding Test, WMS‐R Logical Memory I, RBANS Figure Copy, RBANS Figure Recall, WAIS‐III Digit Span, WAIS‐III Digit Symbol Coding, Phonemic Fluency (FAS), Categorical Fluency, Trail Making Test A and Trail Making Test B. For each task a standardized score (z) was calculated. The z‐score for each task was then combined to create the overall cognition composite. The individual test scores were further grouped into specific cognitive domains—executive function, working memory, processing speed, language, visuospatial function, and visual memory and verbal episodic memory domains based on a priori knowledge (Sherman et al. 2020) and have been described previously (Aleksic et al. 2024).

#### 4.1.2 Atherosclerosis Risk in Communities (ARIC)

The ARIC study(ClinicalTrials.gov identifier: NCT00005131) is an ongoing community‐based cohort study that originally enrolled 15,792 participants beginning in 1987–1989 from four United States communities: Washington County, MD; Forsyth County, NC; northwestern suburbs of Minneapolis, MN; and Jackson, MS (Wright et al. 2021). We used Visit 5 as the baseline for the cross‐sectional analyses and the longitudinal analyses of incident dementia. A total of 5195 participants had SomaScan proteomics (v4.0) measured at Visit 5 (baseline) from stored blood samples. The follow‐up visits included in this analysis are Visits 6 (2016–2017), 7 (2018–2019), and 8 (2020). For analyses adjusting for age, sex, and a composite variable of race and study center, we excluded self‐identified non‐Black and non‐White participants and self‐identified Black participants at Washington County and Minneapolis study sites due to small sample sizes (*n* = 29). Study protocols were approved by Institutional Review Boards at each participating center. All participants gave written informed consent at each study visit, and legally authorized representatives provided consent for participants lacking capacity.

#### 4.1.3 Baltimore Longitudinal Study of Aging (BLSA)

The BLSA (ClinicalTrials.gov identifier: NCT00233272) is a prospective cohort study that enrolled participants mostly residing in the Baltimore‐Washington DC area (Shock 1984). The BLSA recruits participants free of major chronic diseases (except for controlled hypertension) and cognitive or functional disabilities. Once enrolled, participants are followed at varying intervals based on their age (4 years for participants younger than 60 years, every 2 years between 60 and 79 years, and every year for participants older than 80 years). Demographic characteristics including age, sex, and years of education are obtained in a structured interview by study staff. Self‐reported race was grouped into three categories of White, Black, and, due to very small numbers, other races (American Indian or Alaska Native, Chinese, Filipino, Japanese, Hawaiian, other Asian or Pacific Islander, not classifiable, or other non‐white) were grouped together. This analysis included 654 participants with data on plasma proteomics and FI. The study protocol (Protocol number 03‐AG‐0325) was approved by the National Institutes of Health Intramural Research Program Institutional Review Board, and informed consent was obtained from participants at each visit.

### 4.2 Frailty

The two most commonly used operationalizations of frailty are the frailty phenotype or physical frailty (Fried et al. 2001) and cumulative deficit index (Mitnitski et al. 2004; Rockwood and Mitnitski 2007). The present study used the cumulative deficit index proposed by Rockwood et al. as the primary frailty definition (Searle et al. 2008). The variables selected for the FI construction were based on standardized criteria that includes: association with health status, accumulates with age, biologically relevant, and must represent multiple organ systems (Searle et al. 2008). Further, variables should not saturate early with age like presbyopia, which are quite common by age 55. A minimum of 30 variables is recommended for developing the FI (Rockwood and Mitnitski 2007), and has been shown to predict deteriorating health status, institutionalization, and death (Rockwood and Mitnitski 2007). Based on the recommended approach, 41 variables were included in the LonGenity study (Rockwood and Mitnitski 2007). In case of binary variables, 0 represents no deficit and 1 represents a deficit. Continuous or rank variables were graded from 0 (no deficit) to 1 (maximum deficits). The variables and cut‐off used for construction of FI are shown in Table S4. The FI was calculated by adding the score across variables and dividing the sum by the total number of variables per participant; resulting in a range of scores from 0 to 1 for each individual (Rockwood and Mitnitski 2007). For ease of interpretation; the FI was multiplied by 100 (range 0–100) for the analysis, and risk for each 0.01 increase in the FI score reported in statistical analysis. FI showed similar distribution to that obtained in earlier studies (Searle et al. 2008). FI in ARIC (at Visit 5) and BLSA was created using variables as summarized in Tables S5 and S6, respectively. Frailty phenotype in ARIC and BLSA was also operationalized using physical frailty definition defined based on weakness, slowness, exhaustion, weight loss, and low physical activity (Fried et al. 2001; Liu et al. 2023). Details regarding cut points used for each of the five criteria have been provided in previous studies (Landino et al. 2021; Liu et al. 2023; Liu et al. 2024). The presence of no criteria was defined as robust:1–2 criteria as prefrail: and 3–5 criteria as frail.

### 4.3 Mortality

Frequent interactions with participants and family made it possible to track life events well in the LonGenity cohort. In LonGenity, the death of participants was ascertained from designated contacts and supplemented by obituary and National Death Index (NDI) searches by study staff in participants who were lost to follow‐up. For LonGenity participants, mortality data were collected through December 31, 2023.

Deaths were identified in the ARIC study through multiple sources, including semi‐annual follow‐up telephone calls to participants or their proxies, surveillance of local hospitals, state records, and linkage to the National Death Index (NDI). For ARIC participants, mortality data were collected through December 31, 2021. In BLSA, mortality ascertainment of inactive participants was done by telephone follow‐up, correspondence from relatives, and annual searches of the National Death Index through June 7,2021. All‐cause mortality was defined as death from any cause.

### 4.4 Dementia Assessment and Cognitive Scores in the ARIC Study

The primary analysis included 4588 participants (mean age: 75; [SD 5]; 58% women). Participants underwent a comprehensive cognitive and functional assessment at visit 5 and three subsequent visits (Visits 6–8). This included a comprehensive neuropsychological assessment with 10 cognitive measures to assess memory, language, and processing speed and executive function, and an informant interview, as described previously (Knopman et al. 2016). A detailed list of the cognitive measures has been published previously (Knopman et al. 2016). Domain‐specific cognitive scores were obtained from the following tests at Visit 5; (i) the memory domain included the Delayed Word Recall (DWR), the Logical Memory Test (LMT), and the Incidental Learning (ILR); (ii) the language domain included the Animal Naming (AN), the Boston Naming Test (BNT), and the Word Fluency Test (WFT); and (iii) the executive function domain included the Digit Symbol Substitution (DSS), the Trial Making Test A and B (TMTA, TMTB), and the Digit Span Backwards (DSB) (Schneider et al. 2019). Global cognitive scores utilized all the tests above. The methods to generate the domain‐specific and global factor scores were described previously (Gross et al. 2015).

Based on cognitive and functional information collected from participants who attended study visits, dementia was classified using an algorithm and confirmed by an expert committee of physicians and neuropsychologists based on the National Institute on Aging and Alzheimer's Association (McKhann et al. 2011) and the Diagnostic and Statistical Manual of Mental Disorders (Fifth Edition) (American Psychiatric Association and American Psychiatric Association 2013; Knopman et al. 2016). Between visits, participants were contacted for annual and semi‐annual phone interviews in which participants received the six‐item screener (SIS), a brief cognitive assessment (Carpenter et al. 2011). If the participant received a low score on the SIS (or was not able to participate in the screening via phone), the Ascertain Dementia 8‐Item Informant Questionnaire (ad8) (Galvin et al. 2006) was administered to the participant's informant. For participants classified as having dementia based on the cognitive and functional assessment administered at the study visits, these measures were used to estimate the date of dementia onset. For participants who did not attend study visits and were lost to phone follow‐up or died, hospital discharge codes and death certificate codes were used to define dementia diagnoses and date of dementia onset. The analysis of dementia risk in ARIC included participants without dementia at baseline (Visit 5, 2011‐13) and considered incident dementia cases occurring up to December 31, 2020 (Walker et al. 2021).

### 4.5 Ascertainment of Other Variables

Age and sex were based on self‐report in all studies.

#### 4.5.1 LonGenity

Self‐report of physician diagnoses of depression, diabetes, heart failure, hypertension, myocardial infarction, strokes, Parkinson disease (PD), chronic obstructive lung disease, cancer, and arthritis were used in the LonGenity cohort. A Jamar handgrip dynamometer was used to objectively measure dominant hand grip strength at baseline. Gait speed (cm/s) was measured using an 8.5 m long computerized walkway with embedded pressure sensors (GAITRite; CIR Systems, PA). BMI was calculated using the following formula: BMI = weight in kilograms divided by height in meters squared. A BMI of 30.0 or higher was considered as obesity (Sathyan et al. 2024).

#### 4.5.2 ARIC

Hypertension was defined as a systolic blood pressure > 140 mmHg, a diastolic blood pressure > 90 mmHg, or use of hypertensive medication. Diabetes was defined as fasting glucose ≥ 126 mg/dL, non‐fasting glucose ≥ 200 mg/dL, current use of diabetes medication, or self‐reported physician diagnosis. Coronary heart disease (CHD) was defined as a self‐reported history, medical record evidence of myocardial infarction, coronary artery bypass graft or angioplasty, or myocardial infarction determined by ECG adjudication. Heart failure was identified by adjudicated events, ICD code, self‐report physician diagnosis, and self‐report medication use. Stroke was defined as self‐report physician diagnosis at Visit 1. At all subsequent visits, stroke was defined by having had stroke at Visit 1 and adjudicated events after Visit 1. Total cholesterol, HDL, triglyceride, and fasting glucose were measured from plasma or serum. Systolic and diastolic blood pressures were measured three times, and the average of the last measures was used. Grip strength was assessed in the participant's preferred hand using an adjustable, hydraulic grip strength dynamometer. The best of two trials was used for analysis. Gait speed was measured using time to walk 4 m at usual pace.

#### 4.5.3 BLSA

Prevalent cases of cancer were determined based on self‐reports, and excluded basal and squamous cancer. Hypertension was determined based on self‐report, and use of antihypertensive medication, or systolic blood pressure ≥ 140 or diastolic blood pressure ≥ 90. Diabetes was based on fasting glucose ≥ 126 mg/dL or taking diabetes medication. Heart disease was based on self‐reported presence of heart attack, MI, chest pain due to heart disease, coronary bypass surgery or CAGB, angioplasty of coronary artery and taking medication (Nitroglycerin, Beta‐Blocker Agent [BBA] and Calcium Channel Blocker [CCB]). Heart failure was determined based on self‐report; sleeping with 2 or more pillows to breathe and taking diuretic, ACE inhibitor, digoxin, and nitrates; rales at bases or more than normal with presence of edema; or ejection fraction ≤ 40%. Gait speed was assessed in a 6‐m walk at usual pace, and grip strength was measured using a hand‐held dynamometer.

### 4.6 Proteomic Assessment

LonGenity study used 5 k SomaScan Assay V4, which had 5284 SOMAmer reagents, with 5209 SOMAmer reagents targeting human proteins and remaining markers consisting of 22 non‐human proteins, 12 hybridization control elution, 10 non‐biotin, 4 non‐cleavable, 7 deprecated proteins, and 20 spuriomers. SomaScan data standardization was carried out as previously described (Candia et al. 2017). After implementing QC checks, 960 sequences that failed QC were removed. After exclusion of non‐human proteins, deprecated markers, non‐cleavable, non‐biotin, as well as spuriomers, 4265 SOMAmer reagents were available for the proteomic analysis (Sathyan, Ayers, Gao, Milman, et al. 2020; Sathyan, Ayers, Gao, Barzilai, et al. 2020). ARIC used the same version of assay (Liu et al. 2023; Liu et al. 2024). The assay has been shown to have good reproducibility and comparable sensitivity to conventional immunoassay approaches (Walker et al. 2021). Proteomic profiling in BLSA was conducted using the 7k SomaScan assay v4.1, following a protocol that has been detailed elsewhere (Candia et al. 2022). The proteins, expressed as relative fluorescence units, were natural logged transformed before analysis.

### 4.7 Statistical Analysis

#### 4.7.1 Construction of Proteomic Frailty Index

The first step of the study was to investigate the association between the expression levels of 4265 proteins and the cumulative frailty index in a cohort of 880 participants in LonGenity cohort (Sathyan, Ayers, Gao, Milman, et al. 2020). All models were adjusted for age, sex, and parental longevity. To address multiple testing, Bonferroni correction was applied, with a significance threshold set at *p* < 1.17 × 10^−5^ (0.05/4265). In total, 143 proteins were significantly associated with frailty, of which 55 showed positive associations and 88 showed negative associations (Table S7).

The proteomic frailty predictor was constructed using a penalized regression model with the glmnet R package (Friedman et al. 2010). A total of 440 participants in the same cohort were selected for the training set through stratified random sampling. Participants were chosen from each of the frailty score strata (0.00–0.03, 0.03–0.06, 0.06–0.09, 0.09–0.12, 0.12–0.15, 0.15–0.18, 0.18–0.21, …). The remaining participants in the cohort were used to create the validation set.

The FI was regressed on these 143 log‐transformed protein abundances. The cv.glmnet function was used to select the optimal lambda value based on 10‐fold cross‐validation within the training set. An alpha value of 0.5 was set for elastic net regression, representing an equal balance between L1 and L2 regularization. A lambda value (lambda.min parameter) of 0.01019 was selected based on 10‐fold cross‐validation performed on the training set using the cv.glmnet function.

Using elastic net regression on the training data, 25 proteins were selected out of these 143 proteins for the construction of the pFI using the following equation:

$$\begin{array}{cc} \mathrm{pFI}\;=\; & 0.7113+\left(-0.0498^{*}\text{NDST1}\right)+\left(-0.0478^{*}\text{ERBB1}\right)\\ & +\left(-0.0268\times \text{PTGD2}\right)+\left(-0.0199\times \mathrm{GDF}-11/8\right)\\ & +\left(-0.0118\times \text{GLRX3}\right)+\left(-0.0092\times \text{NELL1}\right)\\ & +\left(-0.0089\times \mathrm{CA}2\text{D3}\right)+\left(-0.0084\times \text{ENPP5}\right)\\ & +\left(-0.0079\times \text{VAT1}\right)+\left(-0.0073\times \text{LIRA4}\right)\\ & +\left(-0.0063\times \text{ANTR2}\right)+\left(-0.0033\times \text{WFKN2}\right)\\ & +\left(-0.0023\times \text{Amyloid}-\text{like protein}\;1\right)\\ & +\left(-0.0003\times \text{SEPR}\right)+\left(0.0014\times \text{RNase}\;1\right)\\ & +\left(0.0016\times \text{ATOX1}\right)+\left(0.0025\times \text{FSTL3}\right)\\ & +\left(0.0036\times \text{Troponin}\;T\right)+\left(0.0093\times \mathrm{MIC}-1\right)\\ & +\left(0.0099\times \text{Leptin}\right)+\left(0.0131\times \text{FABPA}\right)\\ & +\left(0.0138\times \mathrm{IL}-\text{1Ra}\right)+\left(0.0218\times \text{COSA1}\right)\\ & +\left(0.0224\times \text{ATL2}\right)+\left(0.0373\times \text{HTRA1}\right) \end{array}$$

Coefficients for each selected protein and the intercept were then used to construct the proteomic frailty index (pFI) for the validation set of the LonGenity cohort, which included 440 participants from LonGenity, the 5195 participants from Visit 5 in the ARIC, and the 654 participants in BLSA. For ease of interpretation, similar to FI, pFI was multiplied by 100 (range 0–100) for the analysis, and risk for each 0.01 increase in the pFI score was reported. A higher pFI score indicated a more frail state, consistent with the traditional FI.

We also performed association analyses of the selected 25 proteins with the measured frailty index in the ARIC cohort, adjusting for age, sex, and a composite variable of race and study center (Table S8).

#### 4.7.2 Association Analyses With pFI

Logistic regression analysis examined the association between the pFI measure and dichotomous disease phenotypes. Robust multiple linear regression using the “lmrob” R function from the “robustbase” package was used to access association between pFI and clinical, physical and cognitive measures. Difference in pFI by physical frailty category (robust versus prefrail and frail) was estimated using a linear regression model.

#### 4.7.3 pFI and Incident Outcomes

##### 4.7.3.1 Mortality

Cox proportional hazard models were used to compute HRs with 95% CIs to predict incident mortality based on pFI. Models were adjusted for age and sex in the LonGenity cohort and additionally for the race‐center composite variable in ARIC and race in BLSA. In all cohorts, participants were categorized into tertiles based on their pFI scores, representing low (T1), medium (T2), and high (T3) proteomic frailty levels. Associations between pFI tertiles and health outcomes were assessed to evaluate gradient effects. Cox proportional hazard models were used to compute HRs with 95% CIs to predict death based on the pFI measure at baseline in LonGenity and BLSA, and visit 5 in the ARIC study. For BLSA, which is a rolling enrollment study, “baseline” was the first visit where data on frailty and proteomics were available.

##### 4.7.3.2 Dementia

Cox proportional hazard models were used to compute HRs with 95% CIs to predict incident dementia based on pFI in ARIC study.

Statistical analyses were conducted using R and analysis in ARIC was conducted using Stata/SE 18.0 (College Station, TX).

## Author Contributions

Sanish Sathyan, Fangyu Liu, Toshiko Tanaka, Luigi Ferrucci, Josef Coresh, Nir Barzilai, Sofiya Milman, Keenan A. Walker, and Joe Verghese contributed to the conception and design of the study. Sanish Sathyan, Fangyu Liu, Toshiko Tanaka, Luigi Ferrucci, Emmeline Ayers, B. Gwen Windham, Erica F. Weiss, Josef Coresh, Julián Candia, Nir Barzilai, Sofiya Milman, Keenan A. Walker, and Joe Verghese contributed to the acquisition of data. Sanish Sathyan, Fangyu Liu, Toshiko Tanaka, B. Gwen Windham, Emmeline Ayers, Sofiya Milman, Keenan A. Walker, and Joe Verghese contributed to the analysis and interpretation of the data. Nir Barzilai, Luigi Ferrucci, Sofiya Milman, Josef Coresh, Keenan A. Walker, and Joe Verghese secured funding and provided supervision. Sanish Sathyan and Fangyu Liu drafted the manuscript, and all authors contributed to the critical revisions of the manuscript. All the authors approved the final version of the manuscript and agree to be accountable for all aspects of the work.

## Conflicts of Interest

Dr. Coresh has served on the SomaLogic scientific advisory committee during 2022–2023. The remaining authors declare no competing interests.

## Supporting information


**Figure S1.** ROC curves for model containing pFI and FI with mortality. AUC of the pFI and FI model were 0.82 without accounting for age and sex.
**Figure S2.** Distribution of pFI and correlation with computed frailty index in ARIC at Visit 5.
**Figure S3.** Distribution of pFI and correlation with computed frailty index in BLSA.
**Table S1.** Function details of possible functionality of the proteins in the pFI.
**Table S2.** Clinical characteristics of ARIC at visit 5.
**Table S3.** Clinical characteristics of BLSA.
**Table S4.** Health variables used for construction of cumulative frailty index in the LonGenity Cohort.
**Table S5.** Health variables used for construction of cumulative frailty index in the ARIC study.
**Table S6.** Health variables used for construction of cumulative frailty index in the BLSA study.

**Table S7.** Complete list of 143 associated proteins associated with frailty phenotype in LonGenity Cohort.
**Table S8.** Association analysis of 25 proteins with FI in ARIC and BLSA.

## Data Availability

LonGenity proteomic data used in this study are available upon request. Please contact the corresponding author for further information. Consistent with a prespecified policy for access of ARIC data, requests may be submitted to the ARIC steering committees for review. BLSA data is available upon request and proposal and submit a pre‐analysis plan for approval (https://blsa.nia.nih.gov/how‐apply).
