# Supplementary material for: A Frailty‐Based Plasma Proteomic Signature Capturing Overall Health and Well‐Being in Older Adults
Source: Aging Cell. 2025 Aug 4;24(9):e70144. doi: 10.1111/acel.70144 (PMC12419846; doi:10.1111/acel.70144)
Supplement: Supplementary file 1 — Figure S1. ROC curves for model containing pFI and FI with mortality. AUC of the pFI and FI model were 0.82 without accounting for age and sex. Figure S2. Distribution of pFI and correlation with computed frailty index in ARIC at Visit 5. Figure S3. Distribution of pFI and correlation with computed frailty index in BLSA. Table S1. Function details of possible functionality of the proteins in the pFI. Table S2. Clinical characteristics of ARIC at visit 5. Table S3. Clinical characteristics of BLSA. Table S4. Health variables used for construction of cumulative frailty index in the LonGenity Cohort. Table S5. Health variables used for construction of cumulative frailty index in the ARIC study. Table S6. Health variables used for construction of cumulative frailty index in the BLSA study. [file ACEL-24-e70144-s002.docx]

**SUPPLEMENTARY FIGURES**

**Supplementary Figure 1:** ROC curves for model containing pFI and FI with mortality. AUC of the pFI and FI model were 0.82 without accounting for age and sex.

**
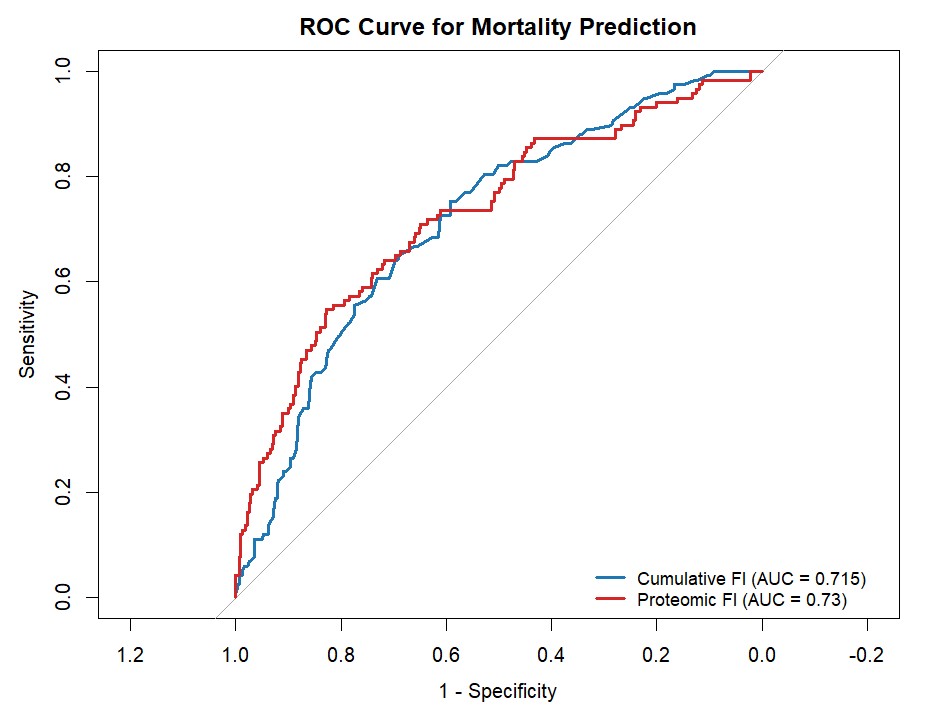
**

**Supplementary Figure 2:** Distribution of pFI and correlation with computed frailty index in ARIC at Visit 5


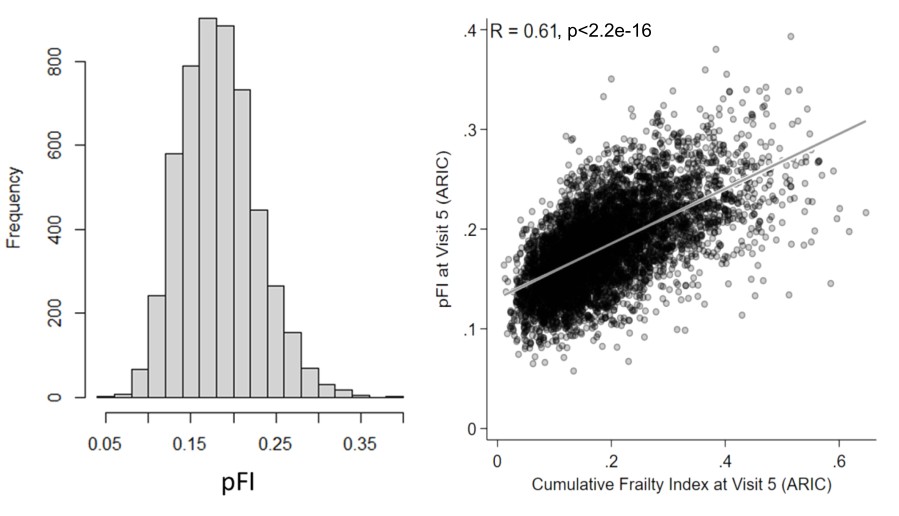


**Supplementary Figure 3:** Distribution of pFI and correlation with computed frailty index in BLSA

**
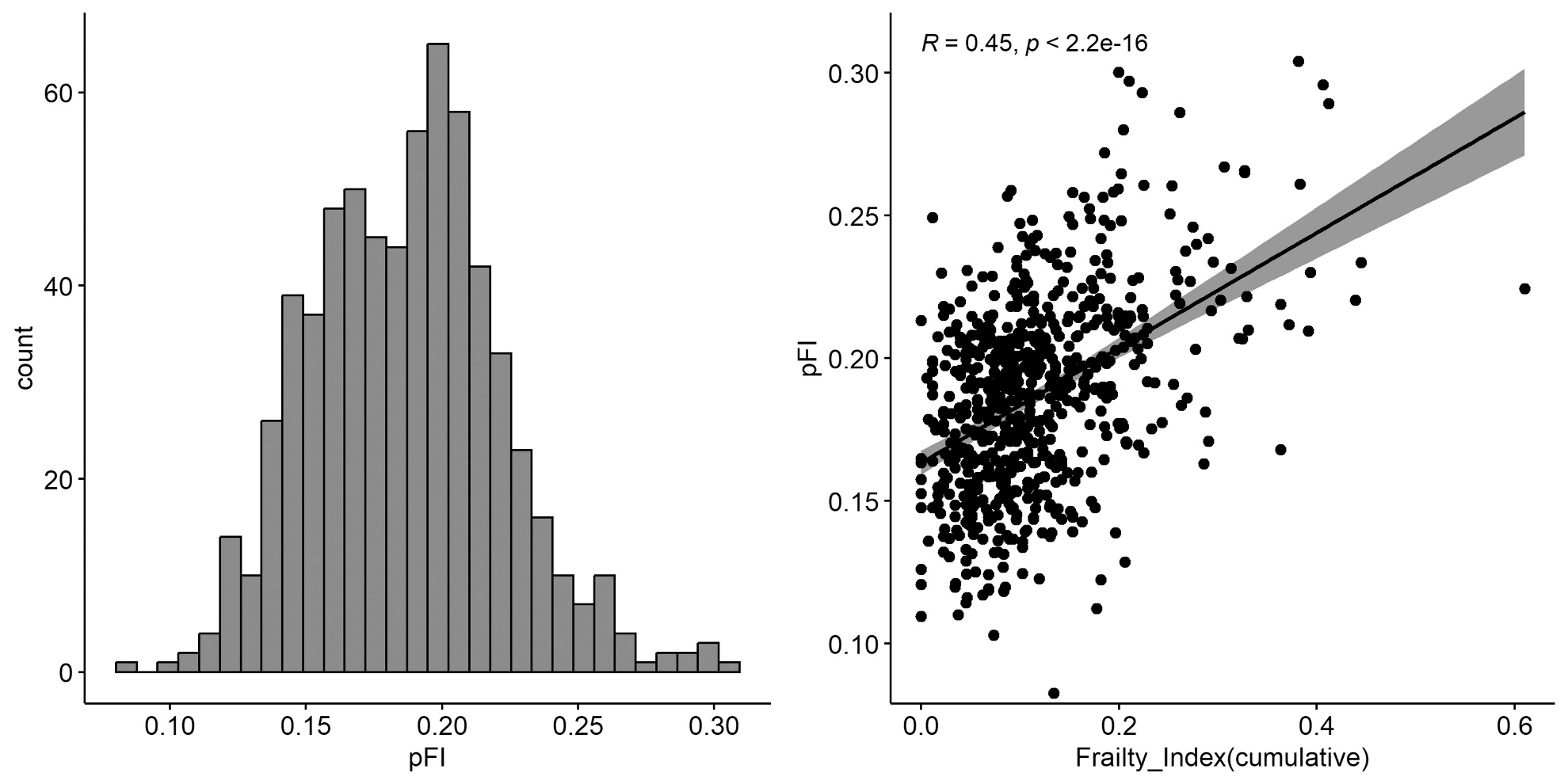
**

**Supplementary Tables**

**Supplementary Table 1: Function details of possible functionality of the proteins in the pFI**

| Directionality | Target | Target Full name | Functionality |
| --- | --- | --- | --- |
| - | NDST1 | Bifunctional heparan sulfate N-deacetylase/N-sulfotransferase 1 | Biosynthesis of heparan sulfate, a glycosaminoglycan critical for cell signaling, development, cognitive function and tissue repair (Khosrowabadi et al., 2024; Whitelock & Melrose, 2011) |
| - | ERBB1 | Epidermal growth factor receptor (EGFR) | Transmembrane protein activated by binding of its specific ligands, including epidermal growth factor and transforming growth factor alpha (TGF-α). EGFR signaling cascade is a key regulator in cell proliferation, differentiation, division, survival, and cancer development (Sabbah, Hajjo, & Sweidan, 2020) |
| - | PTGD2 | Hematopoietic prostaglandin D synthase | Catalyzes the conversion of prostaglandin H2 (PGH2) to prostaglandin D2 (PGD2). PGD2 biological actions include vasodilatation, bronchoconstriction, inhibition of platelet aggregation, and recruitment of inflammatory cells (Kanaoka & Urade, 2003) |
| - | GDF-11/8 | Growth/differentiation factor 11/8 | GDF11 is a circulating TGF-β family member that declines with age. Restoration of youthful GDF11 levels reverses age-related cardiac hypertrophy (Loffredo et al., 2013) |
| - | GLRX3 | Glutaredoxin-3 | Cellular redox regulation and protection against oxidative stress (Ogata, Branco, Vale, & Coppo, 2021) |
| - | NELL1 | Protein kinase C-binding protein NELL1 | Lack of Nell-1 expression is associated with skeletal under mineralization (James et al., 2015) |
| - | CA2D3 | Voltage-dependent calcium channel subunit alpha-2/delta-3 | Calcium channel modulation and regulates many calcium-related biological processes linked to cell growth. SNPs in the gene associated with frailty (Inglés et al., 2019) |
| - | ENPP5 | Ectonucleotide pyrophosphatase/phosphodiesterase family member 5 | Implicated in vascular aging and apoptosis. senescence-associated secretory phenotype factor (Takaya & Kishi, 2024) |
| - | VAT1 | Synaptic vesicle membrane protein VAT-1 homolog | VAT1 is downregulated in aged skin(Sathyan et al., 2020) and also found to be human mammary epithelial cells -specific senescence biomarker (Delfarah, Hartel, Zheng, Yang, & Graham, 2021) |
| - | LIRA4 | Leukocyte immunoglobulin-like receptor subfamily A member 4 | Functions as a coreceptor to limit immune responses to viral infections acting as negative regulator of TLR7 and TLR9 signaling cascades (Cao et al., 2006) |
| - | ANTR2 | Anthrax toxin receptor 2 | ANTXR2 gene coding for ANTR2 protein associated with hypertension (Park et al., 2014; Tikkanen et al., 2018) In mortality prediction modelingANTXR2 was the top negatively associated protein in predicting nervous system-related deaths (Eiriksdottir et al., 2021) |
| - | WFKN2 | WAP, Kazal, immunoglobulin, Kunitz and NTR domain-containing protein 2 | Regulates TGFβ activity in muscle.  Higher protein levels were associated with lower levels of fasting insulin, triglycerides, HOMA-IR and visceral fat, suggesting a protective role against metabolic dysregulation (Ruffieux et al., 2020). |
| - | Amyloid-like protein 1 | Amyloid-like protein 1 | APLP1 is a synaptic cell adhesion molecule that supports the maintenance of dendritic spines and basal synaptic transmission (Schilling et al., 2017) |
| - | SEPR | Prolyl endopeptidase FAP | specifically cleave bonds formed by a proline residue |
| + | RNase 1 | Ribonuclease pancreatic | Upregulated as a protective mechanism during sepsis and cancer (Sun, Han, & Sheng, 2022) |
| + | ATOX1 | Copper transport protein ATOX1 | Atox1 expression is upregulated in injured vessel (Kohno et al., 2013). It plays a role in Cu-induced cell growth. |
| + | FSTL3 | Follistatin-related protein 3 | Binds to and inhibits activins, impacting muscle growth, inflammation, and reproductive functions. Associated with hearth failure (Kizer et al., 2024) |
| + | Troponin T | Troponin T, cardiac muscle | Cardiac muscle contraction regulation; a key biomarker for heart damage, myocardial infraction (Katus et al., 1991) |
| + | MIC-1 | Growth/differentiation factor 15 | Elevated levels are linked to frailty and reduced longevity (Sathyan et al., 2020; Tanaka et al., 2018; Wiklund et al., 2010) |
| + | Leptin | Leptin | Regulates appetite, energy balance and lipid metabolism (Friedman, 2019) |
| + | FABPA | Fatty acid-binding protein, adipocyte | Lipid metabolism, insulin sensitivity, and inflammation. important adipokine linking obesity, inflammation and CVD (Xu et al., 2006) |
| + | IL-1Ra | Interleukin-1 receptor antagonist protein | immune responses and inflammation. IL-1ra is a powerful prognostic marker of mortality in very old people (Jylhä et al., 2007) |
| + | COSA1 | Collagen alpha-1(XXVIII) chain | Extracellular matrix integrity. Associated with incident and prevalent physical frailty (Ramonfaur et al., 2024) |
| + | ATL2 | ADAMTS-like protein 2 | The extracellular matrix glycoprotein ADAMTSL2 is increased in heart failure and inhibits TGFβ signalling in cardiac fibroblasts (Rypdal et al., 2021) |
| + | HTRA1 | Serine protease HTRA1 | HTRA1 cleaves ECM proteins, regulate the availability of insulin-like growth factors (IGFs) by cleaving IGF-binding proteins and has been suggested as a regulator of cell growth (Tiaden & Richards, 2013) |

REFERENCE

Cao, W., Rosen, D. B., Ito, T., Bover, L., Bao, M., Watanabe, G., . . . Liu, Y.-J. (2006). Plasmacytoid dendritic cell–specific receptor ILT7–FcεRIγ inhibits Toll-like receptor–induced interferon production. *The Journal of experimental medicine, 203*(6), 1399-1405.

Delfarah, A., Hartel, N. G., Zheng, D., Yang, J., & Graham, N. A. (2021). Identification of a proteomic signature of senescence in primary human mammary epithelial cells. *Journal of Proteome Research, 20*(11), 5169-5179.

Eiriksdottir, T., Ardal, S., Jonsson, B. A., Lund, S. H., Ivarsdottir, E. V., Norland, K., . . . Holm, H. (2021). Predicting the probability of death using proteomics. *Communications biology, 4*(1), 758.

Friedman, J. M. (2019). Leptin and the endocrine control of energy balance. *Nature metabolism, 1*(8), 754-764.

Inglés, M., Mas-Bargues, C., Gimeno-Mallench, L., Cruz-Guerrero, R., García-García, F. J., Gambini, J., . . . Viña, J. (2019). Relation between genetic factors and frailty in older adults. *Journal of the American Medical Directors Association, 20*(11), 1451-1457.

James, A. W., Shen, J., Zhang, X., Asatrian, G., Goyal, R., Kwak, J. H., . . . Turner, A. S. (2015). NELL-1 in the treatment of osteoporotic bone loss. *Nature communications, 6*(1), 7362.

Jylhä, M., Paavilainen, P., Lehtimäki, T., Goebeler, S., Karhunen, P. J., Hervonen, A., & Hurme, M. (2007). Interleukin-1 receptor antagonist, interleukin-6, and C-reactive protein as predictors of mortality in nonagenarians: the vitality 90+ study. *The Journals of Gerontology Series A: Biological SciencesMedical Sciences, 62*(9), 1016-1021.

Kanaoka, Y., & Urade, Y. (2003). Hematopoietic prostaglandin D synthase. *Prostaglandins, leukotrienes, and essential fatty acids, 69*(2-3), 163-167.

Katus, H. A., Remppis, A., Neumann, F. J., Scheffold, T., Diederich, K. W., Vinar, G., . . . Kuebler, W. (1991). Diagnostic efficiency of troponin T measurements in acute myocardial infarction. *Circulation, 83*(3), 902-912.

Khosrowabadi, E., Mignon-Ravix, C., Riccardi, F., Cacciagli, P., Desnous, B., Sigaudy, S., . . . Molinari, F. (2024). Loss of NDST1 N-sulfotransferase activity is associated with autosomal recessive intellectual disability. *Human molecular genetics, 33*(6), 520-529.

Kizer, J. R., Patel, S., Ganz, P., Newman, A. B., Bhasin, S., Lee, S.-J., . . . Psaty, B. M. J. T. J. o. G. S. A. (2024). Circulating Growth Differentiation Factors 11 and 8, Their Antagonists Follistatin and Follistatin-Like-3, and Risk of Heart Failure in Elders. *The journals of gerontology. Series A, Biological sciences and medical sciences, 79*(1), glad206.

Kohno, T., Urao, N., Ashino, T., Sudhahar, V., McKinney, R. D., Hamakubo, T., . . . Fukai, T. (2013). Novel role of copper transport protein antioxidant-1 in neointimal formation after vascular injury. *Arteriosclerosis, thrombosis, and vascular biology, 33*(4), 805-813.

Loffredo, F. S., Steinhauser, M. L., Jay, S. M., Gannon, J., Pancoast, J. R., Yalamanchi, P., . . . Shadrach, J. L. (2013). Growth differentiation factor 11 is a circulating factor that reverses age-related cardiac hypertrophy. *Cell, 153*(4), 828-839.

Ogata, F. T., Branco, V., Vale, F. F., & Coppo, L. (2021). Glutaredoxin: Discovery, redox defense and much more. *Redox biology, 43*, 101975.

Park, S. Y., Lee, H.-J., Ji, S.-M., Kim, M. E., Jigden, B., Lim, J. E., & Oh, B. (2014). ANTXR2 is a potential causative gene in the genome-wide association study of the blood pressure locus 4q21. *Hypertension Research, 37*(9), 811-817.

Ramonfaur, D., Buckley, L. F., Arthur, V., Yang, Y., Claggett, B. L., Ndumele, C. E., . . . Floyd, J. S. (2024). High Throughput Plasma Proteomics and Risk of Heart Failure and Frailty in Late Life. *JAMA cardiology, 9*(7), 649–658.

Ruffieux, H., Carayol, J., Popescu, R., Harper, M.-E., Dent, R., Saris, W. H., . . . Valsesia, A. (2020). A fully joint Bayesian quantitative trait locus mapping of human protein abundance in plasma. *PLoS computational biology, 16*(6), e1007882.

Rypdal, K. B., Erusappan, P. M., Melleby, A. O., Seifert, D. E., Palmero, S., Strand, M. E., . . . Hubmacher, D. (2021). The extracellular matrix glycoprotein ADAMTSL2 is increased in heart failure and inhibits TGFβ signalling in cardiac fibroblasts. *Scientific reports, 11*(1), 19757.

Sabbah, D. A., Hajjo, R., & Sweidan, K. (2020). Review on epidermal growth factor receptor (EGFR) structure, signaling pathways, interactions, and recent updates of EGFR inhibitors. *Current topics in medicinal chemistry, 20*(10), 815–834.

Sathyan, S., Ayers, E., Gao, T., Weiss, E. F., Milman, S., Verghese, J., & Barzilai, N. (2020). Plasma proteomic profile of age, health span, and all‐cause mortality in older adults. *Aging Cell, 19*(11), e13250. doi:10.1111/acel.13250

Schilling, S., Mehr, A., Ludewig, S., Stephan, J., Zimmermann, M., August, A., . . . Müller, U. C. (2017). APLP1 is a synaptic cell adhesion molecule, supporting maintenance of dendritic spines and basal synaptic transmission. *Journal of Neuroscience, 37*(21), 5345-5365.

Sun, D., Han, C., & Sheng, J. (2022). The role of human ribonuclease A family in health and diseases: A systematic review. *iScience, 25*(11), 105284.

Takaya, K., & Kishi, K. (2024). Regulation of ENPP5, a senescence-associated secretory phenotype factor, prevents skin aging. *Biogerontology*, 1-14.

Tanaka, T., Biancotto, A., Moaddel, R., Moore, A. Z., Gonzalez‐Freire, M., Aon, M. A., . . . Fantoni, G. (2018). Plasma proteomic signature of age in healthy humans. *Aging Cell, 17*(5), e12799.

Tiaden, A. N., & Richards, P. J. (2013). The emerging roles of HTRA1 in musculoskeletal disease. *The American journal of pathology, 182*(5), 1482-1488.

Tikkanen, E., Gustafsson, S., Amar, D., Shcherbina, A., Waggott, D., Ashley, E. A., & Ingelsson, E. (2018). Biological insights into muscular strength: genetic findings in the UK Biobank. *Scientific reports, 8*(1), 6451.

Whitelock, J., & Melrose, J. (2011). Heparan sulfate proteoglycans in healthy and diseased systems. *Wiley interdisciplinary reviews. Systems biology and medicine, 3*(6), 739-751.

Wiklund, F. E., Bennet, A. M., Magnusson, P. K., Eriksson, U. K., Lindmark, F., Wu, L., . . . Pedersen, N. L. (2010). Macrophage inhibitory cytokine‐1 (MIC‐1/GDF15): a new marker of all‐cause mortality. *Aging Cell, 9*(6), 1057-1064.

Xu, A., Wang, Y., Xu, J. Y., Stejskal, D., Tam, S., Zhang, J., . . . Lam, K. S. (2006). Adipocyte fatty acid–binding protein is a plasma biomarker closely associated with obesity and metabolic syndrome. *Clinical chemistry, 52*(3), 405-413.

**Supplementary Table 2. Clinical characteristics of ARIC at visit 5**

| **Variables** | **ARIC visit 5** |
| --- | --- |
|  | N = 5195 |
|  | Mean (SD) / N (%) |
| Age | 75.7 (5.2) |
| Sex |  |
| Men | 2225 (42.8%) |
| Women | 2970 (57.2%) |
| Race-center |  |
| Minneapolis White participants | 1593 (30.7%) |
| Jackson Black participants | 889 (17.1%) |
| Washington White participants | 1465 (28.2%) |
| Forsyth Black participants | 77 (1.5%) |
| Forsyth White participants | 1142 (22.0%) |
| Other race-center combinations | 29 (0.6%) |
| Cumulative frailty index | 0.19 (0.10) |
| Missing | 1 |
| Physical frailty |  |
| Robust | 2174 (41.8%) |
| Prefrail | 2226 (42.8%) |
| Frail | 322 (6.2%) |
| Missing | 473 (9.1%) |
| Memory domain factor score, z-score | 0.0 (0.9) |
| Missing | 57 |
| Executive functioning domain factor score, z-score | 0.0 (0.9) |
| Missing | 119 |
| Language domain factor score, z-score | 0.0 (0.9) |
| Missing | 59 |
| Global cognition factor score, z-score | 0.0 (0.9) |
| Missing | 47 |
| Hypertension |  |
| No | 1358 (26.1%) |
| Yes | 3769 (72.6%) |
| Missing | 68 (1.3%) |
| Diabetes |  |
| No | 3393 (65.3%) |
| Yes | 1656 (31.9%) |
| Missing | 146 (2.8%) |
| CHD |  |
| No | 4315 (83.1%) |
| Yes | 790 (15.2%) |
| Missing | 90 (1.7%) |
| Heart failure |  |
| No | 4510 (86.8%) |
| Yes | 685 (13.2%) |
| Stroke |  |
| No | 4996 (96.2%) |
| Yes | 191 (3.7%) |
| Missing | 8 (0.2%) |
| Total Cholesterol, SI Units | 4.7 (1.1) |
| HDL Cholesterol, SI Units | 1.3 (0.4) |
| V5 Triglycerides, SI Units | 1.4 (0.7) |
| Fasting Glucose, SI Units | 6.3 (1.6) |
| Missing | 216 |
| Systolic Blood Pressure, mmHg | 130.1 (18.1) |
| Missing | 5127 |
| Diastolic Blood Pressure, mmHg | 66.1 (10.7) |
| Missing | 27 |
| Grip strength, kg | 29.1 (10.4) |
| Missing | 411 |
| V5 gait speed (m/sec), mean (SD) | 0.9 (0.2) |
| Missing | 431 |

**Supplementary Table 3. Clinical characteristics of BLSA**

| Variables | BLSA |
| --- | --- |
| n | 654 |
| Age (years) | 76.92 (7.24) |
| Sex (Male) | 313 (47.9) |
| Race |  |
| White | 483 (73.9) |
| Black | 134 (20.5) |
| Other | 37 (5.7) |
|  |  |
| Education (years) | 17.11 (2.73) |
| Smoking |  |
| Current | 19 (2.9) |
| Former | 275 (42.3) |
| Non-smoker | 356 (54.8) |
| Frailty Index | 0.12 (0.08) |
| Cancer | 92 (14.1) |
| Hypertension | 400 (61.2) |
| Diabetes | 91 (13.9) |
| Stroke | 85 (13.0) |
| Heart Failure | 46 (7.0) |
| Heart Disease | 73 (11.2) |
|  |  |
| Walking Speed (m/s) | 1.11 (0.25) |
| Maximum grip strength (kg) | 29.35 (9.91) |
| Systolic blood pressure (mmHg) | 117.09 (15.26) |
| Diastolic blood pressure (mmHg) | 64.34 (8.80) |
| Fasting glucose (mg/dL) | 91.03 (17.13) |
| Triglycerides (mg/dL) | 97.36 (45.73) |
| Total Cholesterol (mg/dL) | 184.85 (36.81) |
| LDL Cholesterol (mg/dL) | 102.91 (32.08) |
| HDL Cholesterol (mg/dL) | 62.47 (17.26) |
|  |  |
| Fasting glucose (SI) | 5.05 (0.95) |
| Triglycerides (SI) | 1.10 (0.52) |
| Total Cholesterol (SI) | 4.79 (0.95) |
| LDL Cholesterol (SI) | 2.66 (0.83) |
| HDL Cholesterol (SI) | 1.62 (0.45) |
|  |  |
| Follow-up time | 7.06 (3.36) |
| Death | 129 (22.2) |

**Supplementary Table 4: Health variables used for construction of cumulative frailty index in the LonGenity Cohort**

| Sl. No. | Variables | Coding |  |
| --- | --- | --- | --- |
| 1 | Help bathing | Yes = 1, No = 0 |  |
| 2 | Help dressing | Yes = 1, No = 0 |  |
| 3 | Help getting in/out of chair | Yes = 1, No = 0 |  |
| 4 | Help walking around house | Yes = 1, No = 0 |  |
| 5 | Help eating | Yes = 1, No = 0 |  |
| 6 | Help grooming | Yes = 1, No = 0 |  |
| 7 | Help using toilet | Yes = 1, No = 0 |  |
| 8 | Help up/down stairs | Yes = 1, No = 0 |  |
| 9 | Help lifting 10 lb | Yes = 1, No = 0 |  |
| 10 | Help shopping | Yes = 1, No = 0 |  |
| 11 | Help with housework | Yes = 1, No = 0 |  |
| 12 | Help with meal preparations | Yes = 1, No = 0 |  |
| 13 | Help taking medication | Yes = 1, No = 0 |  |
| 14 | Help with finances | Yes = 1, No = 0 |  |
| 15 | Lost more than 10 lb in last year | Yes = 1, No = 0 |  |
| 16 | Self rating of health | Poor = 1, Fair = 0.75, Good = 0.5, Very Good = 0.25, Excellent = 0 |  |
| 17 | How health has changed in last year | Worse = 1, Better/Same = 0 |  |
| 18 | Hospitalized/ER visits | Yes = 1, No = 0 |  |
| 19 | Cut down on usual activity (in last month) | Yes = 1, No = 0 |  |
| 20 | Walk outside | <3 days = 1, ≥ 3 days = 0 |  |
| 21 | Feel everything is an effort | Most of time = 1, Sometimes = 0.5, Rarely = 0 |  |
| 22 | Feel depressed | Most of time = 1, Sometimes = 0.5, Rarely = 0 |  |
| 23 | Feel happy | Most of time = 0, Sometimes = 0.5, Rarely = 1 |  |
| 24 | Health interfered with social activities | Not at all - Slightly = 0, Moderately - Extremely = 1 |  |
| 25 | Have trouble getting going | Most of time = 1, Sometimes = 0.5, Rarely = 0 |  |
| 26 | Moderate activity affected | Yes = 1, No = 0 |  |
| 27 | High blood pressure | Yes = 1, No = 0 |  |
| 28 | Heart attack | Yes = 1, No = 0 |  |
| 29 | CHF | Yes = 1, No = 0 |  |
| 30 | Stroke | Yes = 1, No = 0 |  |
| 31 | Cancer | Yes = 1, No = 0 |  |
| 32 | Diabetes | Yes = 1, No = 0 |  |
| 33 | Arthritis | Yes = 1, No = 0 |  |
| 34 | Chronic Lung Disease | Yes = 1, No = 0 |  |
| 35 | Cognitive test: Blessed | <2=0: 2-3=0.25: 4-7=0.50: >7 =1 |  |
| 36 | Peak flow | 1 if Men=<340 liters/min; Women=<310 liters/min |  |
| 37 | BMI | 1 if <18.5 or >=30 |  |
| 38 | Grip strength | 1 if Men BMI=<24, GS=<29: Men BMI 24.1-28, GS=<30: Men BMI>28, GS=<32: |  |
|  |  | Women BMI=<23, GS=<17: Women BMI 23.1-26, GS=<17.3:Women BMI 26.1-29,GS=<18: Women BMI >29, GS=<21 |  |
| 39 | Falls | Yes = 1, No = 0 |  |
| 40 | Memory changes | Yes = 1, No = 0 |  |
| 41 | History of Parkinson's disease | Yes = 1, No = 0 |  |

**Supplementary Table 5: Health variables used for construction of cumulative frailty index in the ARIC study**

| Sl. no | Frailty variables |
| --- | --- |
| 1 | Hypertension |
| 2 | Diabetes |
| 3 | Heart Disease |
| 4 | Stroke |
| 5 | Cancer |
| 6 | Dementia |
| 7 | Chair Stands |
| 8 | Gait Speed |
| 9 | Balance |
| 10 | Everything is an effort |
| 11 | Feel depressed |
| 12 | Feel happy |
| 13 | Feel lonely |
| 14 | Trouble getting going |
| 15 | Hearing problems |
| 16 | Self-rated health |
| 17 | Parkinson Disease |
| 18 | Incontinence |
| 19 | Renal function |
| 20 | Falls |
| 21 | Heart Failure |
| 22 | Chest pain |
| 23 | Weight loss |
| 24 | Smoking |
| 25 | MMSE |
| 26 | No regular exercise |
| 27 | BMI |
| 28 | Grip strength |
| 29 | Atrial fibrillation |
| 30 | Pacemaker |
| 31 | Anemia |
| 32 | Chronic obstructive pulmonary disease, Respiratory disorder |
| 33 | B12 |
| 34 | Thyroid disorder |
| 35 | Difficulty preparing meals |
| 36 | Difficulty dressing |
| 37 | Difficulty walking quarter mile |
| 38 | Difficulty walking up 10 steps without rest |
| 39 | Difficulty stooping, crouching or kneeling |
| 40 | Difficulty lifting or carrying 10 or more pounds |
| 41 | Difficulty doing chores around the house |
| 42 | Difficulty walking from one room to another on the same level |
| 43 | Difficulty standing up from an armless chair |
| 44 | Difficulty getting in or out of bed |
| 45 | Difficulty eating |
| 46 | Seizures |
| 47 | Tremors |
| 48 | Gout |
| 49 | Delusions |
| 50 | Hallucinations |
| 51 | Agitation/aggression |
| 52 | Depression/dysphoria |
| 53 | Anxiety |
| 54 | Apathy |
| 55 | C-reactive protein |
| 56 | Low HDL |
| 57 | Abdominal aortic aneurysm |
| 58 | Albuminuria |
| 59 | Able to do usual activities |
| 60 | Able to walk ½ mile without help |
| 61 | Able to walk up and down stairs without help |
| 62 | Able to do heavy work around the house |
| 63 | Chronic Pain |

**Supplementary Table 6: Health variables used for construction of cumulative frailty index in the BLSA study**

| Sl no | Frailty variables |
| --- | --- |
| 1 | Walking up 10 steps |
| 2 | Lifting and carrying 10lbs |
| 3 | Getting in and out of bed/chairs |
| 4 | Bathing and showering |
| 5 | Dressing |
| 6 | Eating |
| 7 | Using the toilet |
| 8 | Walking across a small room |
| 9 | Doing heavy housework |
| 10 | Preparing your own meals |
| 11 | Shopping for personal items |
| 12 | Using the telephone |
| 13 | Taking medication |
| 14 | Managing finances |
| 15 | Urinary or fecal incontinence |
| 16 | Self-rated health |
| 17 | Feel depressed |
| 18 | Feel everything is an effort |
| 19 | Could not get going |
| 20 | Feel lonely |
| 21 | Feel happy |
| 22 | Orientation to time |
| 23 | Orientation to place |
| 24 | Attention |
| 25 | Recall |
| 26 | Cancer |
| 27 | Anemia |
| 28 | Diabetes |
| 29 | Hypertension |
| 30 | Heart disease, |
| 31 | Congestive heart failure |
| 32 | Stroke |
| 33 | Peripheral artery disease |
| 34 | COPD |
| 35 | Chronic kidney disease |
| 36 | Hip replacement |
| 37 | Joint pain |
| 38 | Depression |
| 39 | Parkinson’s Disease |
| 40 | Cognitive impairment |
| 41 | 5% Unintentional weight loss in the past year |
| 42 | Low physical activity (lowest quartile of physical activity in the past year) |
| 43 | Slow gait speed (lowest quintile walking speed stratified by sex and height) |
| 44 | Weakness (lowest quintile grip strength stratified by sex and BMI) |
